# Periodic health checkups reduce the risk of hospitalization in patients with type 2 diabetes

**DOI:** 10.3389/fcdhc.2023.1087303

**Published:** 2023-01-27

**Authors:** Hidetaka Hamasaki, Hidekatsu Yanai

**Affiliations:** ^1^ Hamasaki Clinic, Kagoshima, Japan; ^2^ Department of Diabetes, Endocrinology, and Metabolism, National Center for Global Health and Medicine, Kohnodai Hospital, Ichikawa, Japan

**Keywords:** periodic health checkup, type 2 diabetes, hospitalization, cardiovascular disease, cost-effectiveness

## Abstract

**Introduction:**

Periodic health checkups (PHCs) represent a unique system in Japan that is useful for the early detection of lifestyle-related diseases and cardiovascular diseases (CVDs). This study aims to investigate the association of PHCs with the hospitalization risk of patients with type 2 diabetes mellitus (T2DM).

**Methods:**

A retrospective cohort study was conducted from April 2013 to December 2015 and included participant information such as CVD history, lifestyle, and whether PHC was conducted in addition to regular medical examinations. Difference in clinical data between patients with and without PHC was examined. Furthermore, Cox regression analysis was performed to investigate the independent association of PHCs with hospitalization.

**Results:**

Herein, 1,256 patients were selected and followed up for 2.35 ± 0.73 years. In the PHC group, body mass index, waist circumference, proportion of patients with a history of CVD, and number of hospitalizations were lower than those in the non-PHC group. Furthermore, the PHC group exhibited a significant association with lower hospitalization risk (hazard ratio = 0.825; 95% confidence interval, 0.684 to 0.997; p = 0.046) in the Cox model.

**Conclusion:**

This study revealed that PHCs minimized the risk of hospitalization in patients with T2DM. Furthermore, we discussed the effectiveness of PHCs in enhancing health outcomes and reducing health care costs in such patients.

## 1 Introduction

Periodic health checkups (PHCs) that are usually conducted annually represent a unique system in Japan created for the early detection of lifestyle-associated diseases and cancer (1). The goal of PHCs is to extend the healthy life span of individuals *via* early detection and treatment of serious ailments, such as cancer and cardiovascular diseases (CVDs), which is the presumed advantage of conducting PHCs. The rate of cancer detection, including that of suspected cancer cases and confirmed cancer diagnoses in examinees, has been estimated as follows: lung cancer, 0.41% and 6.65%; gastric cancer, 0.7% and 11.36%; and breast cancer, 1.34% and 16.23%; respectively (1). However, overdiagnosis or under diagnosis of the target disease constitute a disadvantage of PHCs (1). However, the effectiveness of PHCs from the perspective of public health and health economics remains unclear. Hackl et al. (2) reported that a general health screening program substantially increased both inpatient and outpatient health care costs in the short-term, reduced outpatient health care cost in the medium-term, and demonstrated no effect on both health care cost and health status in examinees in the long-term. A meta-analysis of 17 randomized trials also showed that health checkups exhibited no effect on all-cause mortality, CVD death rate, and the risk of reducing ischemic heart disease and stroke (3). Thus, the overall effectiveness of PHCs is controversial. However, the efficacy of this system also depends on the method and frequency of application and the appropriate selection of individuals (4).

Cost escalation of health care is a serious problem worldwide. The global economic burden of T2DM will increase to >$2 trillion by 2030 (5). The treatment costs of each patient with T2DM and CVDs increase by $3418–$9705 annually compared with patients with T2DM without CVDs (6). The total cost of cancer treatment has inflated faster than cancer incidence among Europeans (7). Hence, it is important to detect such serious diseases at an early stage to prevent disease progression and reduce health care costs.

Patients with T2DM exhibit physically weakness (8), susceptibility to infections (9), high prevalence of CVDs (10), and increased risk of cancer incidence (11); thus, PHCs may contribute to the reduction of health care costs by the early detection and treatment of such serious diseases requiring hospitalization. However, to the best of our knowledge, no studies have investigated the association of PHCs with the hospitalization risk of patients with T2DM. Therefore, this study aims to analyze the association of PHC with the hospitalization risk of T2DM at a regional core hospital in Japan.

## 2 Materials and methods

### 2.1 Study design and subjects

This retrospective cohort study was conducted at National Center for Global Health and Medicine (NCGHM), Kohnodai Hospital, a public, secondary care hospital in Japan. Between April 2013 and December 2015, patients who came to the outpatient department and were diagnosed with T2DM were enquired regarding their medical history. Furthermore, data regarding diabetes and lifestyle-related behaviors after obtaining informed consent from the patients were collected from the Biobank for Metabolic Disorders in the NCGHM Kohnodai Hospital (12) and retrospectively analyzed. As a standard guideline, patient information, such as CVD history, smoking habit, drinking habit, exercise regularity, and whether PHCs were conducted in addition to regular medical examinations, was collected. Regular medical examinations included parameters such as physical measurements of the body, blood pressure (BP) measurement, vision and hearing assessment, routine blood and urine examination, chest X-ray, and electrocardiogram, which were performed at first visit. Patients were excluded if their age was <20 years. Participants were recommended to consume a strict low-calorie diet of 25–30 kcal/kg of ideal body weight at the very first visit by a certified nutritionist. The dietary adherence of the patients was confirmed at every consultation, and the patients followed the diet throughout the research duration. All the participants were assessed and followed up till the end of follow-up up (May 2016) or death. At the end of the follow-up, information regarding hospitalization was collected from the medical record review. The number of hospitalizations was calculated for all the participants. PHCs, which were the primary outcome analyzed, were associated with minimized the hospitalization risk of patients with T2DM. The impact of PHCs on the occurrence of CVDs and all-cause mortality was also examined.

The research design was certified by the Medical Ethics Committee of the NCGHM (Reference No. NCGM-G-002052), this research was conducted as per the Declaration of Helsinki.

### 2.2 PHCs in Japan

PHCs are performed voluntarily and separately from regular medical examinations for patients with T2DM as well as healthy individuals. In Japan, company employees and students have the provision of availing PHCs annually. In addition, Japanese individuals aged between 40 and 74 years are encouraged to undergo annual health checkups that include physical body measurements and examination, BP measurement, blood examination (serum triglycerides, high-density lipoprotein cholesterol, low-density lipoprotein cholesterol, aspartate aminotransferase, alanine aminotransferase, γ-glutamyl transpeptidase, and plasma glucose or hemoglobin A1c [HbA1c]) and urine examination (protein and glucose) to prevent the incidence and progression of lifestyle-associated diseases, such as diabetes mellitus, hypertension, and dyslipidemia (13). Although the Japanese health insurance system does not cover these PHCs, companies and schools cover the costs of the PHCs of their employees and students, respectively. In addition, individuals can avail free PHC services provided by some health insurances schemes (e.g., National Health Insurance). The components of the examinations vary depending on the type of health checkup. For example, a complete comprehensive health checkup known as “Ningen Dock” is conducted in Japan. It includes detailed radiological investigations, such as ultrasonography, computed tomography, magnetic resonance imaging, and endoscopic investigations, in addition to routine blood and urine examinations (1).

### 2.3 Anthropometric values and physiological measurements

A rigid stadiometer (TTM stadiometer; Tsutsumi Co., Ltd., Tokyo, Japan) was employed to document the height of the participants and a standard calibrated scale (AD-6107NW; A&D Medical Co., Ltd., Tokyo, Japan) was used to determine the body weight (BW). Body mass index (BMI) was calculated ***via*** the standard formula: BW (kg) divided by the square of individual height measured (m). Waist circumference (WC) was measured in centimeter using an inch tape at the umbilical level at the end of expiration from a standing position. A standard automatic sphygmomanometer (HBP-9020; Omron Co., Ltd, Tokyo, Japan) was used to measure the BP in a seated position and sedentary state.

### 2.4 CVD history, smoking and drinking habits, and physical activity assessment

A trained professional from the Clinical Research Center of the NCGHM at Kohnodai Hospital recorded the CVD history of the patients, comprising information such as stroke, nonfatal coronary artery or peripheral artery disease, and foul habits, including smoking and alcoholism, during the baseline evaluation. To quantify the smoking habits of the participants, their Brinkman index was calculated as the number of cigarettes smoked per day multiplied by the number of years of smoking (14). Alcohol consumption (AC) was estimated by the type of liquor and amount consumed daily. Furthermore, we enquired regarding the exercise regularity and type, such as jogging, cycling, and calisthenics, of the participants. Using the available information, we calculated the daily exercise duration. Additionally, patients were asked regarding their daily walk-time, except volitional exercise.

### 2.5 Blood examination

We measured the blood glucose and HbA1c of the participants at the time of enrollment. The glomerular filtration rate (eGFR) of the participants was documented using the revised equation exclusively adjusted for the Japanese patients (15).

### 2.6 Sample size

As sample size estimation is very vital for such studies, we employed *post-hoc* sample size estimation using a command comparing survival curves between two independent groups in EZR (16). During this study period, assuming that the hospitalization rates of patients who did and did not undergo PHCs were 38.2% and 48.2%, respectively, the groups were of equal size (1:1 ratio), and the two-tailed level was 0.05. The final sample size was 730 observations needed for a power of 80%. This indicates that the selected sample size had the required power to detect the association between PHCs and hospitalization risk.

### 2.7 Statistical analysis

Data analyses were conducted using SPSS version 25 (IBM Co., Ltd., Chicago, IL). Continuous variables were presented as the mean ± standard deviation (SD). Student’s t test or the Mann–Whitney test, depending on whether the variables followed normal or non-normal distribution, was preferred to find differences between patients who did and did not undergo PHCs, respectively. Categorical variables were expressed as numbers and compared using χ2 test. The Cox proportional hazard equation was used to examine the independent associations of hospitalization, CVD occurrence, and death with PHCs. We included age, gender, BMI, CVD history, AC, Brinkman index for smoking habit, exercise time, systolic BP (SBP), diastolic BP (DBP), blood glucose, HbA1c, and eGFR in the Cox model. The significance level for the study was fixed at <0.05.

## 3 Results

The study recruited 1,256 patients with T2DM, of which 695 were men, 561 were women, and 557 (44.3%) had undergone a PHC. The mean age and BMI were 63.7 ± 13.9 years and 25.5 ± 5.5 kg/m^2^, respectively. Patient characteristics are presented in **Table 1**.

**Table 1:** Demographic characteristics of the participants.

Demographics	
N	1256
Age (years)	63.7 (13.9)
Gender (male/female)	695/561
Exercise time (min/day)	16.0 (45.9)
AC (g ethanol per day)	18.4 (32.3)
Smoking habit (Brinkman index)	329.6 (550.2)
History of CVDs	174
Stroke	91
Myocardial infarction	92
Peripheral artery disease	10
Duration of T2DM (years)	11.7 (11.0)
Anthropometric data	
BMI (kg/m^2^)	25.5 (5.5)
WC (cm)	92 (13.7)
Physiological and biochemical data	
SBP (mmHg)	131.2 (19.9)
DBP (mmHg)	73.6 (14.2)
Plasma glucose (mg/dL)	159.4 (64.0)
HbA1c (%)	7.5 (1.7)
Estimated glomerular filtration rate (mL/min/1.73 m^2^)	73.0 (23.6)

Data are presented as mean, standard deviation (SD) other than for number of subjects, gender, and history of CVDs.

AC, alcohol consumption; BMI, body mass index; CVDs, cardiovascular diseases; DBP, diastolic blood pressure; HbA1c, hemoglobin A1c; T2DM, type 2 diabetes mellitus; SBP, systolic blood pressure; WC, waist circumference

BMI, WC, number of patients with CVD history, and number of hospitalizations during the study period were lower in the PHC group than in the non-PHC group. Furthermore, AC, exercise time, and walking time were higher in the PHC group than in the non-PHC group. On an average, patients without PHC were admitted once to our hospital during the study period (**Table 2**).

**Table 2:** Comparison of clinical data between patients with and without periodic health checkups.

	With periodic health checkups	Without periodic health checkups	p
N	557	699	–
Age (years)	63.8 (12.9)	63.7 (14.6)	0.99
Gender (Male/Female)	250/307	311/388	0.91
BMI (kg/m^2^)	25.0 (5.2)	25.8 (5.7)	0.013
WC (cm)	90.5 (13.3)	93.2 (14.0)	0.001
Duration of diabetes (years)	11.5 (10.9)	11.9 (11.1)	0.54
AC (g/day in ethanol consumption)	19.3 (33.3)	17.6 (31.5)	0.032
Smoking habits (Brinkman index)	305.6 (523.4)	348.9 (570.5)	0.063
Exercise time (min/day)	18.7 (46.9)	13.9 (15.0)	0.003
Walking time (min/day)	31.4 (47.5)	22.4 (39.9)	<0.001
SBP (mmHg)	131.5 (19.3)	130.9 (20.4)	0.48
DBP (mmHg)	74.4 (14.0)	72.9 (14.3)	0.069
Plasma glucose (mg/dL)	157.5 (64.8)	160.8 (63.4)	0.17
HbA1c (%)	7.5 (1.7)	7.5 (1.8)	0.60
eGFR (mL/min/1.73 m^2^)	74.0 (21.9)	72.1 (24.8)	0.17
History of CVDs (yes/no)	62/495	112/580	<0.001
Number of hospitalizations per patient	0.7 (1.5)	1.0 (2.1)	<0.001

Data are represented as the mean value except for the number of subjects, gender, and history of CVDs.

AC, alcohol consumption; BMI, body mass index; CVDs, cardiovascular diseases DBP, diastolic blood pressure; HbA1c, hemoglobin A1c; eGFR, estimated glomerular filtration rate; SBP, systolic blood pressure; WC, waist circumference

During a mean follow-up of 857 ± 267 days, 20 patients (1.6%) died, 14 (1.1%) sustained cardiovascular events, and 550 (43.8%) were admitted to our hospital. The number of patients who were hospitalized at least once was 213 (38.2%) in the PHC group and 337 (48.2%) in the non-PHC group. The total number of hospitalizations was 1092, of which 382 belonged to the PHC group and 710 to the non-PHC group. Of these, 378 (34.6%) belonged to the diabetes, endocrinology, and metabolism ward, 165 (15.1%) to the surgery ward, 135 (12.4%) to the internal medicine ward, 85 (7.8%) to the ophthalmology ward, 79 (7.2%) to the gastroenterology ward, 65 (6.0%) to the hepatology ward, 36 (7.5%) to the cardiology ward, 27 (2.5%) to the respiratory medicine ward, and 122 (11.1%) to other wards, such as neurology, rheumatology, and psychiatry.

Furthermore, Cox proportional hazard analyses with adjustment for age, gender, BMI, CVD history, drinking habit, smoking habit, exercise time, SBP, DBP, blood glucose, HbA1c, and eGFR showed that PHCs exhibited a significant impact on patient hospitalization risk (hazard ratio [HR] = 0.825; 95% confidence interval [CI], 0.684–0.997; p = 0.046); however, there was no significant association of PHCs with CVDs and all-cause mortality (**Table 3**).

**Table 3:** Cox proportional hazard analysis for evaluating the association of periodic health checkups with hospitalization, CVDs, and all-cause mortality in patients with type 2 diabetes.

	Hospitalization			CVDs			All-cause mortality		
	HR	95% CI	p	HR	95% CI	p	HR	95% CI	p
Age (per 1 year increase)	1.021	1.011–1.031	<0.001	1.112	1.022–1.210	0.014	1.035	0.980–1.093	0.21
Gender									
Male	1.179	0.955–1.457	0.13	1.432	0.389–5.274	0.59	0.347	0.097–1.245	0.10
Female	(reference)			(reference)			(reference)		
BMI (per 1 unit increase in kg/m^2^)	1.019	0.999–1.040	0.063	1.123	0.996–1.266	0.058	0.810	0.696–0.942	0.006
History of CVDs									
Yes	1.304	1.028–1.654	0.029	14.999	4.063–55.375	<0.001	0.276	0.034–2.225	0.23
No	(reference)			(reference)			(reference)		
AC (per 1 g/day increase in ethanol consumption)	1.002	1.000–1.005	0.074	0.997	0.968–1.026	0.81	1.013	1.004–1.022	0.006
Smoking habit (per 1 unit increase in Brinkman index)	1.000	1.000–1.000	0.55	0.999	0.997–1.001	0.21	1.001	1.000–1.002	0.001
Exercise time (per 1 min/day increase)	0.998	0.996–1.000	0.093	1.009	1.000–1.018	0.057	0.994	0.979–1.010	0.49
Systolic blood pressure (per 1 mmHg increase)	1.004	0.998–1.011	0.19	1.012	0.973–1.052	0.56	0.991	0.953–1.030	0.64
Diastolic blood pressure (per 1 mmHg increase)	0.994	0.984–1.003	0.18	1.040	0.972–1.112	0.26	0.995	0.939–1.055	0.88
Plasma glucose (per 1 mg/dL increase)	1.001	1.000–1.003	0.084	0.996	0.982–1.010	0.59	1.010	1.001–1.019	0.027
HbA1c (per 1% increase)	1.340	1.256–1.429	<0.001	1.070	0.633–1.808	0.80	0.832	0.540–1.282	0.41
eGFR (per 1 unit increase in mL/min/1.73 m^2^)	1.001	0.996–1.006	0.61	1.020	0.987–1.055	0.24	0.980	0.956–1.004	0.11
Periodic health checkup									
Yes	0.825	0.684–0.997	0.046	2.297	0.667–7.910	0.19	1.461	0.495–4.316	0.49
No	(reference)			(reference)			(reference)		

AC, alcohol consumption; BMI, body mass index; CI, confidence interval; CVDs, cardiovascular diseases; eGFR, estimated glomerular filtration rate; HbA1c, hemoglobin A1c; HR, hazard ratio; WC, waist circumference

## 4 Discussion

We demonstrated that PHCs can remarkably lower the hospitalization risk of patients with T2DM. Although PHCs were not correlated with CVDs and all-cause mortality, the primary result of the study suggests that PHCs improved health outcomes in patients with T2DM. To the best of our knowledge, this is the first study to demonstrate that all-cause hospitalization could be prevented in patients with T2DM who have undergone a PHC in addition to regular medical examinations.

A previous systematic review and meta-analysis concluded that general PHCs were not beneficial for reducing all-cause mortality and cardiovascular events; however, the studies included were from Western countries (United States, United Kingdom, Denmark, Sweden, Belgium, Poland, Italy, and Montenegro) (3). Ethnic differences should be considered while evaluating the effectiveness of PHCs on health outcomes in patients with T2DM. The average BMI of Japanese individuals with T2DM was 24.8 kg/m² in 2021 (17). The prevalence of a BMI of ≥30 kg/m² varies by country and ranges from 3.7% in Japan to 38.2% in the United States (18). Obese individuals are susceptible to stress-induced eating habits, with a greater preference for high-sugar and high-fat foods (19), which deteriorate metabolic health and results in the development of diseases that require hospitalization. Hence, evidence regarding the effectiveness of PHCs for health in Western countries is not always applicable to Eastern countries such as Japan. In this regard, the findings of the present study are important.

Generally, patients who regularly visit hospitals for chronic diseases undergo PHCs voluntarily in Japan; thus, health consciousness and literacy could also influence their behavior. A systematic review reported that health literacy was a significant determinant of obesity (20). Moreover, adequate health literacy was inversely related to physical inactivity (odds ratio [OR] = 0.48; 95% CI, 0.39–0.59) in individuals with CVDs (21), and patients with diabetes who did not adequately understand health information were sedentary (OR = 3.43; 95% CI, 2.14–5.51) (22). Herein, patients in the PHC group were less obese and more active than patients in the non-PHC group. Furthermore, rate of history of CVD was lesser in the PHC group than in the non-PHC group. These results suggest that patients who underwent PHC exhibited high health literacy and consciously attempted to prevent obesity through increasing daily physical activity, thereby resulting in primary prevention activities against CVDs. Health literacy also constitutes an important factor for the self-management of diabetes in patients with T2DM (23); however, there was no significant difference in HbA1c levels between the groups in this study. The reason behind why chronic glycemic control did not vary between the groups cannot be explained based on the findings of the present study. However, glucose fluctuation, which cannot be assessed by HbA1c levels, appears to encourage the development of cardiovascular events in patients with diabetes than chronic hyperglycemia (24). Furthermore, high plasma glucose levels have been independently associated with inadequate health literacy in patients with T2DM (25). Hence, patients in the non-PHC group could have possibly experienced hypoglycemia and hyperglycemia more often compared with patients in the PHC group. Further studies are warranted to characterize the relationship between PHCs and the rate of health literacy and glycemic control.

A higher AC in the PHC group than in the non-PHC group is inconsistent with the finding that the patients who underwent PHCs were less obese and more active than the patients who did not undergo PHC. However, previous studies have indicated that AC impacted the levels of physical activity in a positive manner (26). Additionally, AC was 19.3 and 17.6 g/day in the PHC and non-PHC groups, respectively, which is not harmful for cancer, CVDs, and mortality in Japanese people (27). Furthermore, even after adjusting AC in the Cox model, PHC was significantly associated with hospitalization risk in this study. Therefore, we propose that PHCs are not directly related to increase in AC.

Treatment costs vary depending on the cause of hospitalization, type and severity of disease, patient condition, and type of health care organization. For example, hospitalization due to uncontrolled diabetes costs ~¥40,000 ($296 at the current exchange rate) per day in our organization (28). Considering that the average length of a hospital stay due to diabetes is 13–14 days in our organization (28), the hospitalization cost is >¥50,0000 ($3,703 at the current exchange rate). Conversely, the cost of PHCs also varies significantly depending on the components of examination. For example, a nationwide annual health checkup to prevent the incidence and progression of lifestyle-associated diseases costs ~¥7,000 ($55 at the current exchange rate) per examination (29), whereas a comprehensive health checkup in our organization, which includes chest X-ray, abdominal ultrasonography, gastroscopy, and some cancer screening tests, in addition to routine blood and urine examinations costs ¥57,000 ($422 at the current exchange rate) for men and ¥64,000 for women ($474 at the current exchange rate) (30). Herein, PHC was equated with a 17.5% reduction in the risk of all-cause hospitalization in patients with T2DM, which is comparable with >¥87,500 ($648 at the current exchange rate) when converted to hospitalization costs due to diabetes. This study is not a randomized controlled trial; however, if the number of patients who would need intervention is calculated as follows: 1/(0.482 − 0.382) = 10, we need to provide PHCs to 10 patients to prevent one additional hospitalization in this study cohort. Thus, comprehensive health checkup may not be cost effective; however, providing a general PHC annually might be cost effective for the managing patients with T2DM who are at a higher risk of hospitalization. Furthermore, the cost of hospitalization due to ischemic heart disease with percutaneous coronary intervention (¥1,100,000; $8,148 at the current exchange rate), acute cerebral infarction (¥1,190,000; $8,814 at the current exchange rate), and cancers, such as lung cancer with chemotherapy (¥610,000; $4,519 at the current exchange rate), are higher than that of hospitalization due to diabetes (28). The recurrence risk of CVDs was significantly high (HR = 14.999; 95% CI, 4.063–55.375; p < 0.001) in this study (**Table 3**). Patients with T2DM exhibit a high prevalence of CVDs (6), and an increased risk of liver, pancreatic, and endometrial cancers (11); therefore, PHCs that focuses on cardiovascular risks and early detection of such cancers may be beneficial for cost-reduction as well as for improving health and life expectancy in such patients.

However, PHC is not necessary for every healthy individual. Current evidence suggests that regular nationwide health checkups are neither beneficial nor cost effective to improve health outcomes (31). Compared with other developed countries, such as the United Kingdom, Canada, and Sweden, PHCs in Japan cover an unusually wide range and high volume of the population, and it is unclear whether all examinations contribute to the overall health of the population and provide financial benefits (31). The target population for PHCs should be narrowed down to ensure that health care providers can effectively and cost-efficiently apply the results of PHCs in primary and secondary prevention activities for CVDs and cancers. In addition, we need to establish nationwide standardized screening programs that include what, when, to whom, and how PHCs should be administered by considering national and international evidence and best practices to efficiently use limited medical resources (31). In this regard, the findings of this study are highly suggestive. At least, we might as well consider the adoption of PHCs as a secondary prevention strategy in the management of patients with T2DM.

Reducing inequities in health and raising health awareness across all social strata are critical for improving health in patients with T2DM. In Japan, company employees and students can avail PHCs every year at the expense of the company or school, respectively. However, the unemployment rate has been positively associated with reduced PHCs (32). Furthermore, women with lower socioeconomic statuses are less likely to avail cancer screening examinations, which cost ¥500−¥1,000 with municipal subsidies (33). Individuals with low socioeconomic statuses generally exhibit a higher risk of smoking, drinking, physical inactivity, poor nutrition, and not undergoing PHCs (32, 34, 35). Therefore, it is also vital that health promotion policies for PHCs target patients with T2DM with low socioeconomic statuses.

There are several limitations of this study that need to be addressed. First, we did not investigate the components of PHCs. Whether or not the participants have undergone a PHC was gathered from their response to the question “Do you have a PHC except regular medical examinations for diabetes?” Hence, information regarding the type of PHC; for example, general health checkup, cancer screening test, or comprehensive PHC, was lacking. Second, we did not assess the detailed causes of the hospitalizations; for example, the name and severity of the disease. We can speculate that a certain number of hospitalizations were related to diabetes because one third of the total hospitalizations were in the diabetes, endocrinology, and metabolism ward; however, how PHCs were related with the heightened risk of hospitalizations due to a specific disease remains unknown. Third, we assessed the adherence of the participants to the diet therapy by asking the question “Do you stick to the diet therapy?” at the regular medical examinations approximately once a month; however, the adherence rate of the diet therapy should be measured *via* a specific tool (e.g., diet diary). Fourth, the socioeconomic status and educational level of the participants were not investigated. Individuals with high risk factors, such as smoking, physical inactivity, and low education and socioeconomic statuses, are less likely to attend PHCs than others (28, 29). Such demographic data should be incorporated in future studies.

## 5 Conclusion

PHCs exhibit a significant role in minimizing the hospitalization risk of patients with T2DM. PHCs may be useful in improving health outcomes of patients and reduce health care costs when targeting individuals with high risk factors, such as T2DM. Health policy makers need to improve PHC programs by including what, when, to whom, and how PHCs should be administered. Furthermore, a discussion should be conducted to reach a consensus regarding how the results of PHCs can be effectively applied to effectively patient management.

## Data availability statement

The raw data supporting the conclusions of this article will be made available by the authors, without undue reservation.

## Ethics statement

The studies involving human participants were reviewed and approved by The Medical Ethics Committee of the National Center for Global Health and Medicine (Reference No. NCGM-G-002052). Written informed consent for participation was not required for this study in accordance with the national legislation and the institutional requirements.

## Author contributions

HH conducted the study, performed the data analyses, drafted the manuscript, and revised the manuscript. HY critically reviewed the manuscript and the scientific interpretations of study results. All authors contributed to the article and approved the submitted version.
